# Tapering towards drug-free remission in rheumatoid arthritis: assessment of clinical outcomes and drug savings

**DOI:** 10.1093/rap/rkaf124

**Published:** 2025-10-24

**Authors:** Thomas Bradley, Jasmine P X Sim, Kenneth F Baker

**Affiliations:** Translational and Clinical Research Institute, Newcastle University, Newcastle upon Tyne, UK; NIHR Newcastle Biomedical Research Centre, Newcastle upon Tyne, UK; Translational and Clinical Research Institute, Newcastle University, Newcastle upon Tyne, UK; NIHR Newcastle Biomedical Research Centre, Newcastle upon Tyne, UK; Translational and Clinical Research Institute, Newcastle University, Newcastle upon Tyne, UK; NIHR Newcastle Biomedical Research Centre, Newcastle upon Tyne, UK; Rheumatology Department, Newcastle upon Tyne Hospitals NHS Foundation Trust, Newcastle upon Tyne, UK

Key message

- Tapering to drug-free remission in rheumatoid arthritis is clinically feasible with potential drug cost savings.

Dear Editor, Around two-thirds of patients with RA can achieve clinical remission with modern treat-to-target regimens of DMARDs, albeit with the risks of long-term immunosuppression and the need for resource-intensive drug monitoring and prescribing systems. Many patients in sustained clinical remission choose to taper DMARDs, with up to 50% achieving drug-free remission (DFR), though predicting this remains challenging [1]. While some clinical characteristics have been linked to the maintenance of remission during tapering, findings vary considerably between trials [2] and outcome data from drug tapering in routine clinical practice are limited. Additionally, although a handful of European studies have investigated the economic outcomes associated with tapering [3], data from UK-based cohorts remain scarce. Further research is needed to clarify the predictability and clinical impact of flares and better characterise the economic effects of DMARD tapering in practice.

We aimed to assess the clinical outcomes and drug savings of DMARD tapering in a cohort of RA patients in remission, particularly the frequency and clinical impact of disease flares, and the drug costs saved by employing standardised DMARD tapering schedules.

Data presented were derived from patients enrolled in the RheumatOid Arthritis DMard tAPering (ROADMAP) service at Newcastle Hospitals between January 2023 and March 2025. The entry criteria for the ROADMAP service were a clinical diagnosis of RA, 28-joint DAS with CRP (DAS28-CRP) <2.4 at enrolment and no escalation of treatment/use of steroid treatment in the past 12 months. Patients enrolled in the ROADMAP service tapered their DMARD therapy at 3-monthly reviews as part of routine clinical care according to a locally agreed DMARD tapering schedule (Supplementary Table S1). Additional patient-requested visits were utilised in the event of a possible flare. Arthritis flare was defined a priori by fulfilment of both a DAS28-CRP score >2.4 or at least one swollen joint and patient or clinician impression of flare. Confirmed flares were treated with the reintroduction of treatment from the previous tapering step and corticosteroid administration if clinically indicated. Patients who did not fulfil the flare definition were categorised as being in remission.

Thirty-seven patients were enrolled in the ROADMAP cohort at the time of analysis (Supplementary Table S2). Data were censored at the last visit before the date of analysis (24 March 2025), with 23 (62%) patients maintaining remission. Of these, 10 were in DFR for a median follow-up of 336 days [interquartile range (IR) 137–495; range 98–679], 6 were continuing to taper and 7 had decided to stop tapering short of DMARD discontinuation. Thirteen patients (35%) met the flare definition during follow-up, at a median of 259 days (IQR 189–300; range 20–650) from enrolment (Fig. 1). One further patient experienced an extra-articular disease flare (persistent pleural effusion) despite their arthritis remaining in remission and was withdrawn from follow-up. In exploratory analyses, ACPA seronegativity was associated with a trend towards an increase in the maintenance of remission during tapering, although not reaching statistical significance (Supplementary Fig. S1). We did not observe a difference in maintenance of remission between patients tapering conventional synthetic *vs* biologic/targeted synthetic DMARDs (Supplementary Fig. S2).

**Figure 1.**
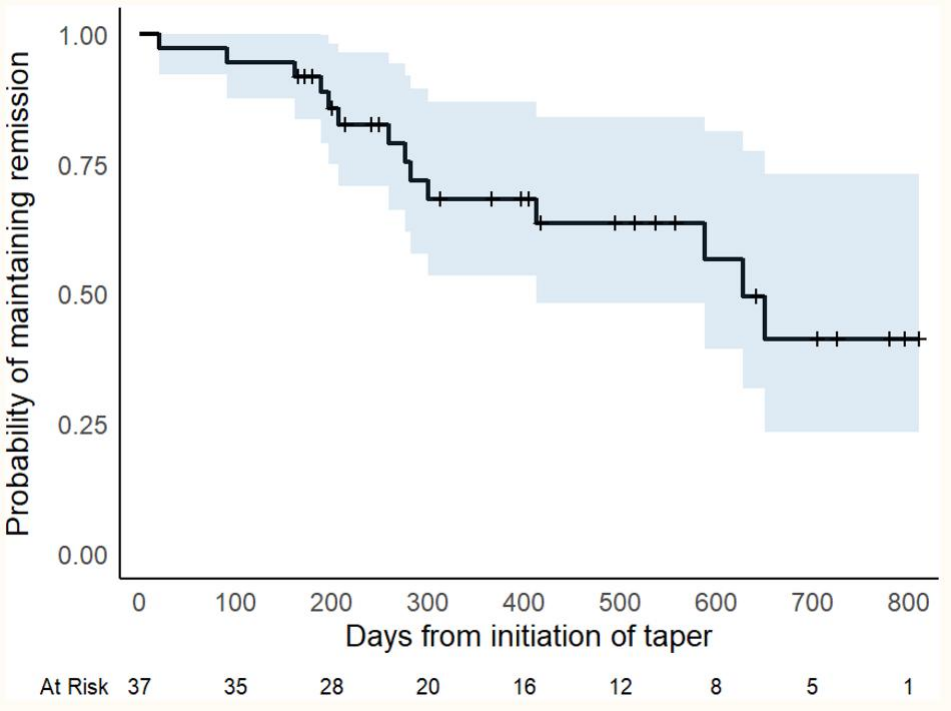
Kaplan–Meier survival curve for the ROADMAP cohort. Shaded area represents the 95% confidence interval

Of the 13 patients who fulfilled the flare definition, DAS28-CRP-based disease activity [4, 5] was high (DAS28-CRP >4.6) (*n* = 4) during one flare, moderate (>2.9–4.6) (*n* = 5) during six flares, low (2.4–2.9) (*n* = 5) during five flares and was classified as remission (DAS28-CRP <2.4) in one case with swollen joints outside of DAS28 assessment. Disease activity increased from baseline at pre-flare and flare visits, falling back to baseline at post-flare visits (Supplementary Fig. S3). However, the median increase in DAS28-CRP at pre-flare visits (0.28) is unlikely to be clinically meaningful. A total of 12/13 patients who fulfilled the flare definition had a post-flare review by the time of analysis, of whom remission was recaptured in all patients with a DMARD dose lower (*n* = 5), equal to (*n* = 6) or greater than (*n* = 1) baseline. Only 2/12 flares spanned more than one visit interval (>3 months). There was a small increase in HAQ Disability Index scores at flare (median increase 0.12) (Supplementary Fig. S4), which returned to baseline within 3 months.

According to prices listed in the British National Formulary, tapering saved a total of £74 633.60 over 27 months (Supplementary Tables S3 and S4). Savings were mostly realised in patients tapering a biologic or targeted synthetic DMARD, in whom tapering saved a median of £4934 per patient per year, although exact drug cost savings will vary with differing local procurement arrangements.

In conclusion, these data present real-world evidence from a UK cohort that tapering to DFR is achievable in some RA patients and where flare occurs it is relatively mild in most cases, with short-lived clinical impact. Nevertheless, our observations are exploratory and further analyses in larger and more representative cohorts are required. While we observed a considerable savings in drug costs, an in-depth health economic analysis is needed to assess wider direct and indirect healthcare costs, quality of life and work productivity together with patient satisfaction measures. Repeated analysis of the ROADMAP service cohort with an increased sample size and follow-up is planned to further validate our findings.

## Supplementary data

Supplementary data are available at *Rheumatology Advances in Practice* online.

## Supplementary Material

rkaf124_Supplementary_Data

## Data Availability

The data underlying this article cannot be shared publicly to maintain the privacy of individuals who participated in the study.
