# Supplementary material for: Tapering towards drug-free remission in rheumatoid arthritis: assessment of clinical outcomes and drug savings
Source: Rheumatol Adv Pract. 2025 Oct 24;9(4):rkaf124. doi: 10.1093/rap/rkaf124 (PMC12624437; doi:10.1093/rap/rkaf124)
Supplement: rkaf124_Supplementary_Data [file rkaf124_supplementary_data.zip › 25-141 Supplementary material.docx]

Supplementary material


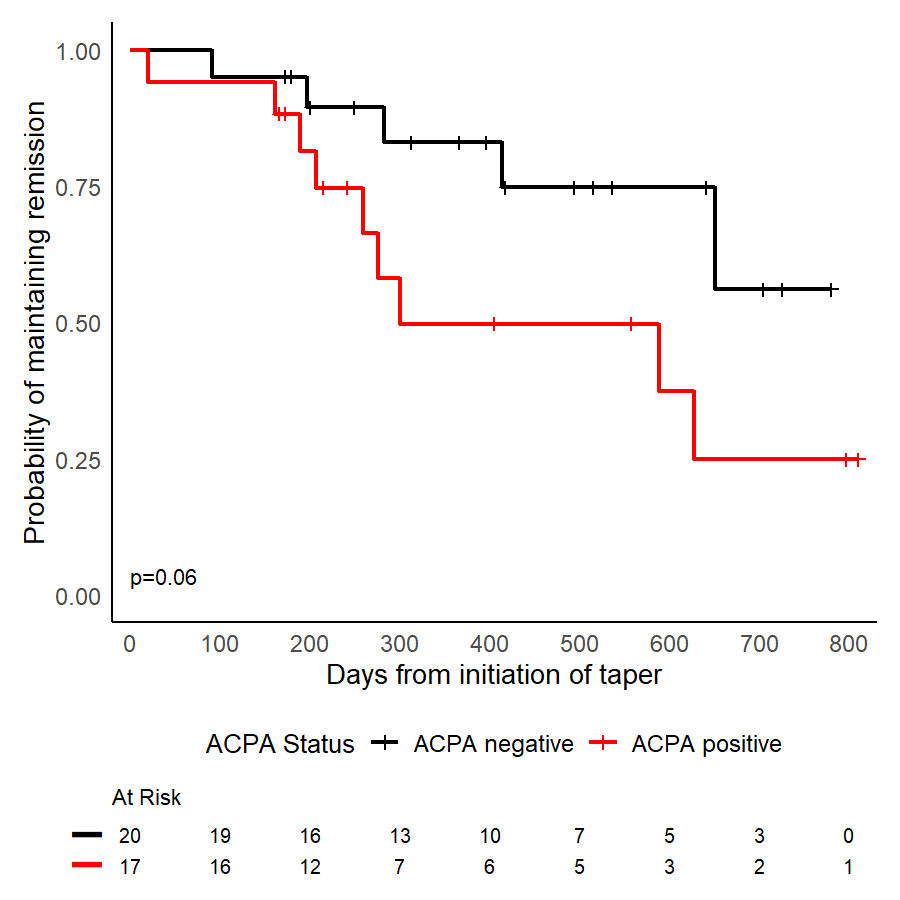


Supplementary Figure 1: Kaplan-Meier survival curve exploratory analysis for the ROADMAP cohort stratified by anti-citrullinated peptide antibody (ACPA) status, showing a statistically non-significant trend towards higher remission rates in the seronegative group (p=0.06, log rank test).


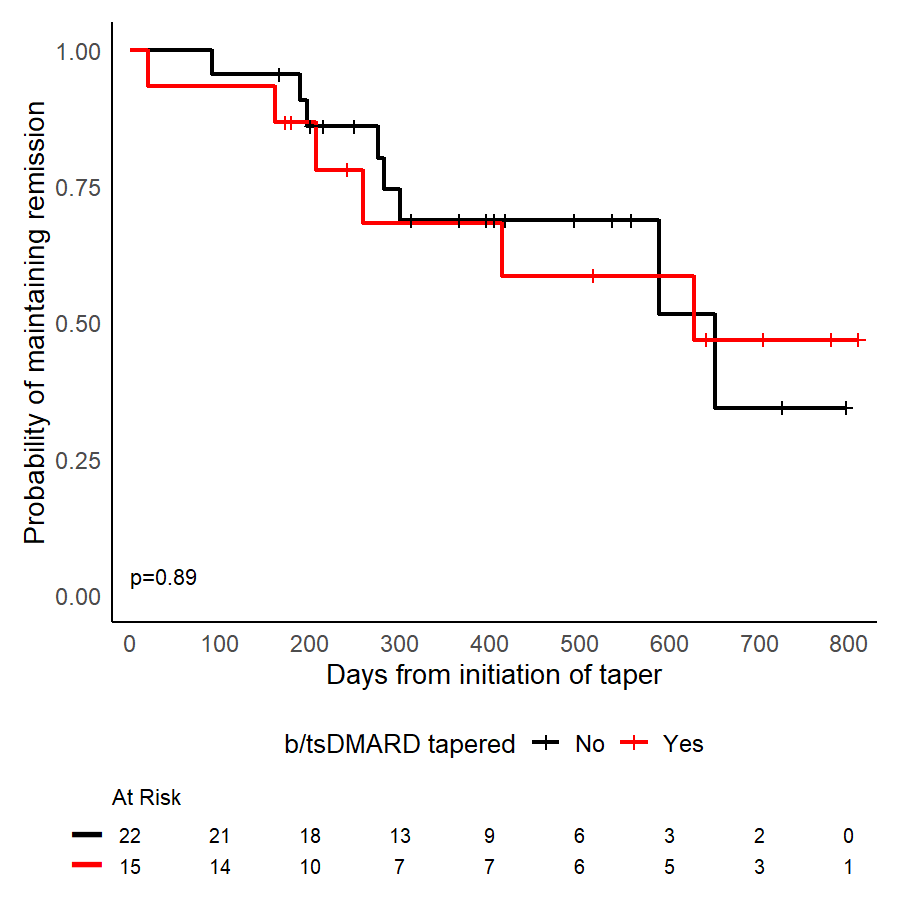


Supplementary Figure S2: Kaplan-Meier survival curve exploratory analysis for the ROADMAP cohort stratified by whether patients tapered a conventional synthetic DMARD (csDMARD) or biologic/targeted synthetic DMARD (b/tsDMARD). No significant difference in remission rates based on DMARD class was observed (p=0.89, log-rank test).


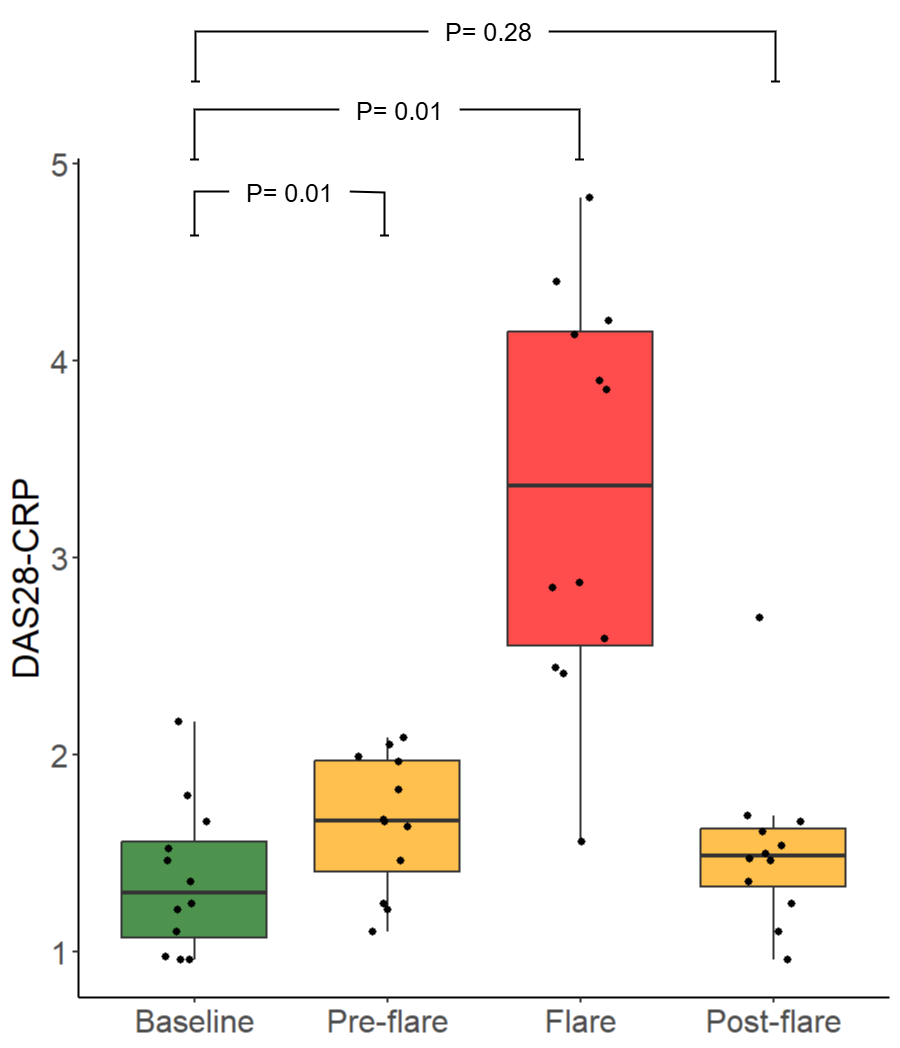


Supplementary Figure S3: Change in DAS28-CRP with visit status in patients who experienced a flare (n = 12). Only patients with data at all time points are displayed. Pre-flare visits were defined as a patient's last visit before a disease flare. A post-flare visit was the first visit following the resolution of a disease flare. For flares spanning multiple visits, DAS28-CRP at the initial flare visit was plotted. Statistical testing (paired Wilcoxon signed-rank test) is presented for illustrative exploratory analysis purposes only. One patient fulfilled the flare definition despite a DAS28-CRP < 2.4 owing to the presence of joint swelling outside of DAS28 assessment. One patient had tonsillitis at post-flare review, causing an elevated CRP and DAS28-CRP score but no signs of active synovitis.


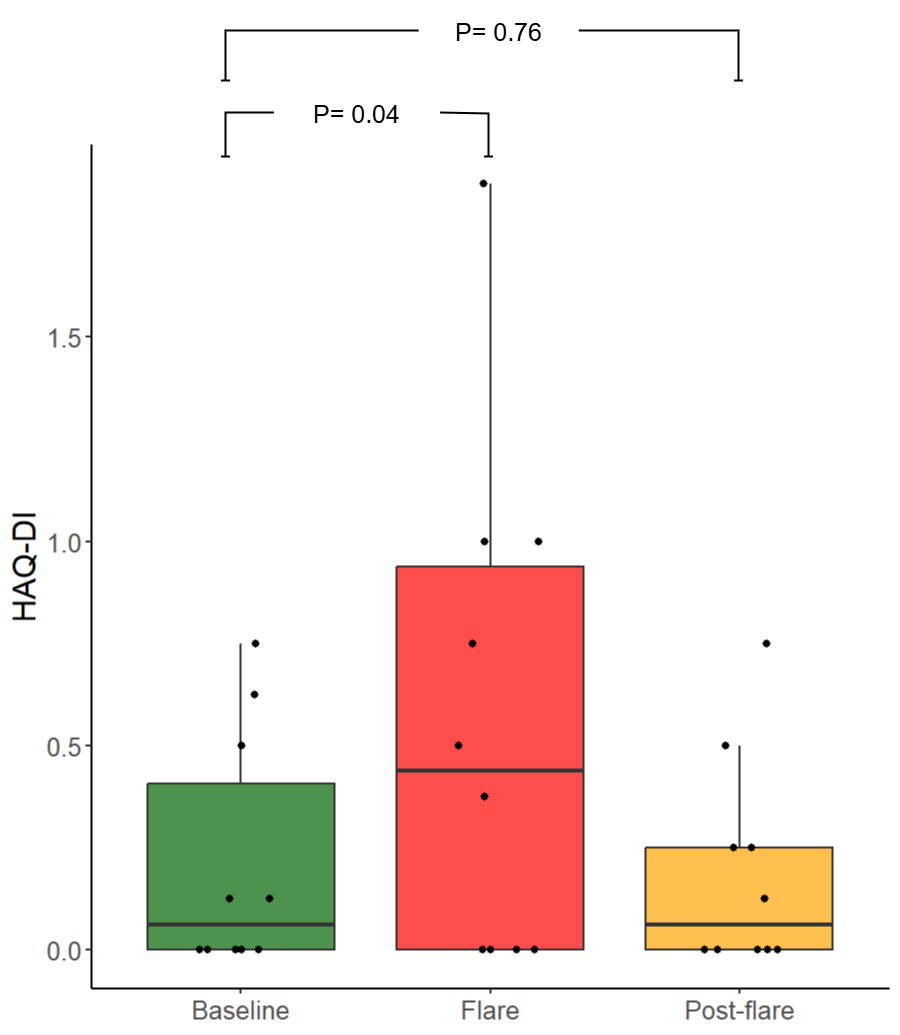


Supplementary Figure S4: Change in HAQ-DI with visit status in flare patients (n= 10). Only patients with data at all time points are displayed. For flares spanning multiple visits, HAQ-DI at the initial flare appointment was plotted. Statistical testing (paired Wilcoxon signed-rank test) is presented for illustrative exploratory analysis purposes only.

Supplementary Table S1: DMARD tapering schedule in ROADMAP, as approved by the Newcastle Hospitals Rheumatology Clinical Governance Group.

| **DMARD** | **Step 1** | **Step 2** | **Step 3** | **Step 4** |
| --- | --- | --- | --- | --- |
| **Methotrexate^1^** | 20mg | 15mg | 10mg | Stop |
| **Sulfasalazine^1^** | 2g daily | 1g daily | Stop | - |
| **Leflunomide** | Stop | - | - | - |
| **Hydroxychloroquine** | Stop | - | - | - |
| **JAK inhibitor** | Half dose tablet^2^ (if available) | Every 2 days | Stop | - |
| **Twice weekly biologic** | Every week | Every 2 weeks | Stop | - |
| **Once weekly biologic** | Every 2 weeks | Every 3 weeks | Every 4 weeks | Stop |
| **Once fortnightly biologic** | Every 3 weeks | Every 4 weeks | Every 6 weeks | Stop |
| **Once monthly biologic** | Every 2 months | Every 3 months | Stop | - |

^1^ Starting at first tapering step below the patient’s prescribed dose at time of enrolment.

^2^ Where a JAK inhibitor is usually prescribed twice daily (e.g. tofacitinib), then once daily dosing may be used as an alternative to halving the dose.

Supplementary Table S2: Baseline demographics of the ROADMAP patient cohort. *Boolean remission is defined as: TJC28 ≤1, SJC28 ≤1, C-reactive protein ≤10 mg/L, PGA≤10 mm. Abbreviations: ACPA; Anti-Citrullinated Peptide Antibody, DAS28-CRP; Disease Activity Score with C-reactive Protein, HAQ-DI; Health-Assessment Questionnaire-disability index, IQR; Inter-quartile range, RADAI-5; Rheumatoid Arthritis Disease Activity Index 5, RhF; Rheumatoid factor.

| **Demographic** | **Patients tapering DMARDs (n=37)** |
| --- | --- |
| **Patient characteristics** | |
| Female: n (%) | 23 (62) |
| Age: median (IQR) [range] | 67 (59-76) [29-91] |
| Height (cm): median (IQR) [range] | 166 (161-172) [153-190] |
| Weight (kg): median (IQR) [range] | 69.5 (65.9-80) [39.50-97.90] |
| Smoking status: current (ex) [never] | 3 (16) [18] |
| Smoking pack years: median (IQR) [range] | 1 (0-10) [0-60] |
| Alcohol consumption (units per week): median (IQR) [range] | 2 (0-10) [0-70] |
| **Disease characteristics** | |
| Years from diagnosis: median (IQR) [range] | 7.8 (5.1-10.4) [2.1-37.8] |
| RhF positive: n (%) | 12 (32) |
| ACPA positive: n (%) | 16 (43) |
| DAS28-CRP at baseline: median (IQR) [range] | 1.36 (0.97-1.66) [0.96-2.48] |
| RADAI-5* at baseline: median (IQR) [range] | 0.6 (0.1-2.2) [0-6.8] |
| CRP at baseline in mg/L: median (IQR) [range] | 1 (0-4) [0-15] |
| ESR at baseline in mg/L: median (IQR) [range] | 9 (3.5-12) [2-73] |
| Boolean remission** at baseline: n (%) | 27 (73) |
| HAQ-DI at baseline***: median (IQR) [range] | 0 (0-0.56) [0-1.5] |
| **DMARD use** | |
| Total number DMARDs ever: median (IQR) [range] | 3 (2-3) [1-5] |
| Ever used biologic: n (%) | 17 (46) |
| Taking >1 DMARD at baseline: n (%) | 22 (59) |
| Tapering methotrexate at baseline: n | 19 |
| Tapering hydroxychloroquine at baseline: n | 3 |
| Tapering sulfasalazine at baseline: n | 2 |
| Tapering etanercept at baseline: n | 5 |
| Tapering adalimumab at baseline: n | 5 |
| Tapering tocilizumab at baseline: n | 1 |
| Tapering leflunomide at baseline: n | 1 |
| Tapering baricitinib at baseline: n | 1 |

Supplementary Table S3: Dose and cost savings for DMARDs tapered via a decrease in dose.

| **Demographic** | **Methotrexate (mg)** | **Sulfasalazine (g)** | **Baricitinib (mg)** | **Hydroxychloroquine (g)** | **Leflunomide (mg)** |
| --- | --- | --- | --- | --- | --- |
| **Dose related** | | | | | |
| Number of patients tapering medication: n | 24 | 3 | 2 | 3 | 1 |
| Total dose of medication saved: n (% of dose if no taper) | 18612 (50.1) | 1613.5 (43.0) | 686 (70.0) | 366.80 (90.0) | 1960 (100.0) |
| Dose of medication saved per patient: median (IQR) [range] | 488 (137- 1076) [65-1930] | 273.0 (267.8-675.5) [262.5-1078.0] | 343 (NA) [133-553] | 134.4 (105.0-145.6) [75.6-156.8] | 1960 (NA) [NA] |
| Dose of medication saved per patient per year: median (IQR) [range] | 484 (228-714) [30-909] | 182.5 (156.0-425.8) [129.4-669.2] | 245(NA) [231-260] | 73.0 (65.5-73.0) [58.0-73.0] | 3650 (NA) [NA] |
| Flares during tapering: n (% of those tapering medication) | 8 (33) | 0 (0) | 1 (50) | 0 (0) | 0 (0) |
| **Cost related** | | | | | |
| Cost of medication: number of tablets (dose) [price] | 28 (Methotrexate 2.5mg tablet) [£1.44] | 112 (Salasopyrin 500mg tablet) [£4.87] | 28 (Olumiant 4mg tablet) [£805.56] | 60 (Hydroxychlroquine 200mg tablet) [£2.96] | 30 (Leflunomide 10mg tablet) [£1.91] |
| Total cost saved | £382.88 | £140.32 | £9,868.11 | £90.48 | £12.47 |
| Cost saved per patient: median (IQR) [range] | £12.34 (£4.09-£22.37) [£1.34-£39.71] | £23.74 (£23.29-£58.74) [£22.83-£93.75] | £2467.03 (NA) [£956.60-£3977.45] | £33.15 (£25.90-£35.91) [£18.65-£38.68] | £12.47 (NA) [NA] |
| Cost saved per patient per year: median (IQR) [range] | £9.96 (£4.69-£14.68) [£0.63-£18.69] | £15.87 (£13.56-£37.03) | £1765.55 (NA) [1662.67-£1868.43] | £18.01 (£16.15-£18.01) [£14.30-£18.01] | 23.24 (NA) [NA] |

Supplementary Table S4: Dose and cost savings for bDMARDs tapered via a decrease in dosing frequency. *One patient increased their etanercept dosing frequency post flare, hence doses/cost saved for this patient were negative.

| **Demographic** | **Etanercept*** | **Adalimumab** | **Tocilizumab** |
| --- | --- | --- | --- |
| **Dose related** | | | |
| Number of patients tapering medication: n | 7 | 5 | 1 |
| Total doses of medication saved: n (% of doses if no taper) | 148 (25.2) | 105 (53.5) | 73 (78.4) |
| Doses of medication saved per patient: median (IQR) [range] | 22 (11-40) [-55-78] | 18 (10-23) [8-48] | 31 (NA) [NA] |
| Doses of medication saved per patient per year: median (IQR) [range] | 15 (7-30) [-24-34] | 14 (9-13) [9-21] | 20 (NA) [NA] |
| Flares during tapering: n (% of those tapering medication) | 4 (57) | 1 (20) | 0 (0) |
| **Cost related** | | | |
| Cost of medication*: number of injections (dose) [price] | 4 (Erelzi 50mg/1ml solution) [£643.50] | 2 (Amgevita 40mg/0.8ml solution) [£633.60] | 4 (RoActerma 162mg/0.9ml solution) [£931.12] |
| Total cost saved | £23,788.60 | £33,366.51 | £6984.23 |
| Cost saved per patient: median (IQR) [range] | £3460.42 (£1837.43-£6494.32)  [£-8779.18-£8616.47] | £3855.19 (£2970.35-£4236.52) [£2376-£15119.62] | £6984.23 (NA) [NA] |
| Cost saved per patient per year: median (IQR) [range] | £2478.02 (1175.49- £4800.54)  [-£3985.57-£5423.79] | £3855.19 (£2970.35- £4236.52) [£2766.91-£6804.76] | £4668.94 (NA) [NA] |
